# Combining carbonic anhydrase and thioredoxin reductase inhibitory motifs within a single molecule dramatically increases its cytotoxicity

**DOI:** 10.1080/14756366.2020.1734800

**Published:** 2020-03-05

**Authors:** Mikhail Krasavin, Tatiana Sharonova, Vladimir Sharoyko, Daniil Zhukovsky, Stanislav Kalinin, Raivis Žalubovskis, Tatiana Tennikova, Claudiu T. Supuran

**Affiliations:** a Saint Petersburg State University, Saint Petersburg, Russian Federation; b Latvian Institute of Organic Synthesis, Riga, Latvia; c Faculty of Materials Science and Applied Chemistry, Institute of Technology of Organic Chemistry, Riga Technical University, Riga, Latvia; d Neurofarba Department, Universita degli Studi di Firenze, Florence, Italy

**Keywords:** Anticancer agents, carbonic anhydrase inhibition, thioredoxin reductase inhibition, synergistic effect, dual pharmacophores, Michael acceptors, zinc-binding group, hypoxia, oxidative stress, cancer cell defence mechanisms

## Abstract

A hypothesis that simultaneous targeting cancer-related carbonic anhydrase *h*CA IX and *h*CA XII isoforms (whose overexpression is a cancer cell’s defence mechanism against hypoxia) along with thioredoxin reductase (overexpressed in cancers as a defence against oxidative stress) may lead to synergistic antiproliferative effects was confirmed by testing combinations of the two inhibitor classes against pancreatic cancer cells (PANC-1). Combining both pharmacophoric motifs within one molecule led to a sharp increase of cytotoxicity. This preliminary observation sets the ground for a fundamentally new approach to anticancer agent design.

## Graphical Abstract

**Figure:**
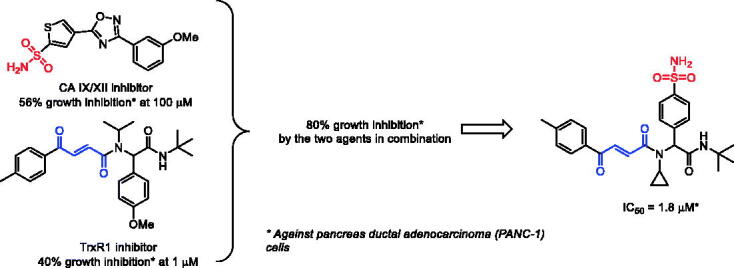

## 1. Introduction

Growing malignant tumours possess numerous defence mechanisms which ensure survival of aberrantly proliferating cancer cells while normal cells in the surrounding tissues are being killed and replaced with the neoplasm.^1^ Among these mechanisms, overexpression of membrane-bound isoforms of carbonic anhydrase (*h*CA IX and XII) protect tumours from hypoxia and provide defence against acidification of the environment surrounding the growing tumour.^2^ Selective targeting of *h*CA IX and XII has been confirmed to lead to retardation of tumour growth and, ultimately, reduction of tumour size.^3^ Recently, we reported on the discovery of potent 1,2,4-oxadiazole-based inhibitors **1**–**3** of these two cancer-related isoforms of carbonic anhydrase.^4^^,^^5^ These compounds possess subnanomolar to double-digit nanomolar potency against *h*CA IX and XII (Table 1), which makes them ideal tool compounds for further investigation of the role of these carbonic anhydrase isoforms in tumour growth.

**Table 1.** Chemical structures and inhibitory profile of carbonic anhydrase inhibitors **1**–**3** and thioredoxin reductase inhibitors **4**–**6** employed in this study (the pharmacophoric inhibitory motifs are highlighted red and blue, respectively).

Compound	Structure	K_i_, nM^5^		TrxR1 IC_50_, μM^12^
		*h*CA IX	*h*CA XII	
**1**		0.089	10.3	–
**2**		2.2	33.6	–
**3**		92.4	8.9	–
**4**		–	–	1.74
**5**		–	–	0.93
**6**		–	–	0.60

Another important mechanism of tumour survival which can be considered a target for anticancer agent design,^6^ is that providing tumour cell defence against oxidative stress (reactive oxygen species or ROS). In particular, tumour cells have been shown to overexpress thioredoxin reductase (TrxR, EC 1.8.1.9) which contributes to their resistant phenotype characterised by higher levels of ROS.^7^ Thus, targeting TrxR1 (the most widespread cytosolic isoform of human TrxR) is an emerging approach to selective killing of cancer cells.^8^ TrxR is a selenocysteine (Sec) enzyme which, along with NADPH and thioredoxin (Trx), constitutes the thioredoxin system responsible for maintaining Trx in the reduced bis-sulfhydryl state.^9^ Among several classes of inhibitors possessing a variable degree of electrophilicity towards the catalytic Sec residue (reviewed by Bellelli^10^ and Fang^11^), we found covalent Michael acceptor inhibitors (such as Ugi-type adducts **4**–**6** which we dubbed “Ugi Michael Acceptors” or UMAs) to be particularly efficacious.^12^ From the mechanistic standpoint, the inhibitory action of UMAs towards TrxR1 likely is exerted *via* the irreversible covalent trapping of the catalytic Sec residue (which exists in the ionised form at physiological pH^13^) by the electrophilic β-benzoyl acrylamide (or acrylate) moiety present in **4**–**6**. The inhibitory potency of these compounds was established at the submicromolar to low micromolar level (Table 1) on a recombinant TrxR1 enzyme and compound **6** (DVD-445) was nominated a lead for further optimisation.

In this work, we aimed at verifying whether combinations of the said inhibitors from different classes (i.e. any of **1**–**3** with any of **4**–**6**) would have stronger antiproliferative effects on tumour cells in comparison with a given single agent. With this idea in mind – along with the general understanding of the pharmacophoric moieties present in the carbonic anhydrase inhibitors (the zinc-binding primary sulphonamide group,^3^ ZBG) and thioredoxin reductase inhibitor (the α,β-unsaturated Michael acceptor moiety^12^) – we also aimed to combine these critical inhibitory motifs in the structure of a single agent and verify whether this will lead to potentiation of its cytotoxicity compared to inhibitors **1**–**6**. Herein, we present the results of these studies.

## 2. Materials and methods

### 2.1. Chemical syntheses – general

All reagents and solvents were obtained from commercial sources and used without purification. All reactions implemented in an open flask without any protection from CO_2_ and H_2_O. Reactions were monitored by analytical thin-layer chromatography (TLC) Macherey-Nagel, TLC plates Polygram^®^ Sil G/UV254. Visualisation of the developed chromatograms was performed by fluorescence quenching at 254 nm. ^1^H and ^13^C NMR spectra were measured on Bruker AVANCE DPX 400 (400 MHz for ^1^H and 100 MHz for ^13^C respectively). All chemical shifts (δ) are given in parts per million (ppm) with reference to solvent residues in DMSO-d_6_ (2.50 for proton and 39.52 for carbon) and coupling constant (*J)* are reported in hertz (Hz). Multiplicities are abbreviated as follows: s = singlet, d = doublet, t = triplet, q = quartette, m = multiplet, br = broad. Melting points were determined on Electrothermal IA 9300 series Digital Melting Point Apparatus. Mass spectra were recorded on microTOF spectrometers (ESI ionisation).

### 2.2. General *Procedure 1*: preparation of compounds 1–2 (GP1)

To a solution of carboxylic acid **7** (1.1 mmol) in dry DMSO (1.0 ml) CDI (195 mg, 1.2 mmol) was added. The reaction mixture was stirred at room temperature for 30 min, and then amidoxime **8** (1.0 mmol) was added. The reaction mixture was stirred at room temperature for another 18 h, then to the reaction mixture powdered NaOH (48 mg, 1.2 mmol) was added rapidly. The reaction mixture was stirred at room temperature for 2 h. Then the reaction mixture was diluted with cold water (20 ml). The resulting precipitate was filtered off, washed with cooled water (15 ml) and dried in air at 50 °C.

#### 2.2.1. 4-(3-Cyclopropyl-1,2,4-oxadiazol-5-yl)thiophene-2-sulphonamide (1)

Yield 203 mg (75%). White solid, m.p. 311–313 °С. ^1^H NMR (400 MHz, DMSO) *δ* ppm 8.72 (d, *J* = 1.6 Hz, 1H), 7.97 (d, *J* = 1.6 Hz, 1H), 7.90 (s, 2H), 2.19 (m, 1H), 1.15 − 1.09 (m, 2H), 1.00 − 0.95 (m, 2H).). ^13^C NMR (101 MHz, DMSO) *δ* ppm 173.0 (C), 170.6 (C), 148.8 (C), 135.5 (CH), 128.5 (CH), 124.8 (C), 8.1 (CH_2_), 6.9 (C). HRMS (ESI, *m/z*): calculated for C_9_H_9_N_3_O_3_S_2_ [M + H]^+^ 272.0158; found 272.0152.

#### 2.2.2. 4-(3-(3-Methoxyphenyl)-1,2,4-oxadiazol-5-yl)thiophene-2-sulphonamide (2)

Yield 222 mg (66%). Yellow solid, m.p. 167–169 °С. ^1^H NMR (400 MHz, DMSO-d_6_) *δ* ppm 8.85 (d, *J* = 1.5 Hz, 1H), 8.10 (d, *J* = 1.5 Hz, 1H), 7.95 (s, 2H), 7.66 (d, *J* = 7.7 Hz, 1H), 7.57 − 7.55 (m, 1H), 7.52 (t, *J* = 8.0 Hz, 1H), 7.20 (dd, *J* = 8.3, 2.6 Hz, 1H), 3.86 (s, 3H).). ^13^C NMR (101 MHz, DMSO-d_6_) *δ* ppm 171.3 (C), 168.5 (C), 160.2 (C), 149.0 (C), 135.9 (CH), 131.0 (CH), 128.6 (CH), 127.7 (C), 124.7 (C), 119.9 (CH), 118.2 (CH), 112.5 (CH), 55.8 (CH_3_). HRMS (ESI, *m/z*): calculated for C_13_H_11_N_3_O_4_S_2_ [M + H]^+^ 338.0264; found 338.0266.

### 2.3. 3-(5-Cyclopropyl-1,2,4-oxadiazol-3-yl) benzenesulphonamide (3)

To a solution of amidoxime **9** (1.0 mmol) and methyl cyclopropanecarboxylate (1.5 mmol) in DMSO (1.0 ml) powdered NaOH (60 mg, 1.5 mmol) was rapidly added. The reaction mixture was stirred at room temperature for 2 h. The reaction mixture was diluted with cold water (20 ml). The resulting precipitate was filtered off, washed with water (15 ml) and dried in air at 50 °C.

Yield 151 mg (57%). Beige solid, m.p. 249–251 °С. ^1^H NMR (400 MHz, DMSO-d_6_) *δ* ppm 8.40 (s, 1H), 8.17 (d, *J* = 6.7 Hz, 1H), 8.00 (d, *J* = 8.2 Hz, 1H), 7.76 (t, *J* = 7.7 Hz, 1H), 7.53 (s, 2H), 2.47 − 2.40 (m, 1H), 1.36 − 1.27 (m, 2H), 1.24 − 1.18 (m, 2H). ^13^C NMR (101 MHz, DMSO-d_6_) *δ* ppm 182.8 (C), 167.1 (C), 145.6 (C), 130.6 (CH), 130.4 (CH), 128.8 (CH), 127.4 (C), 124.6 (CH), 10.7 (CH), 7.7 (CH_2_). HRMS (ESI, *m/z*): calculated for C_11_H_11_N_3_O_3_S [M + H]^+^ 266.0594; found 266.0618.

### 2.4. General procedure for the preparation of compounds 4–6

Aldehyde (1 mmol) and amine (1 mmol) were combined in methanol (3 ml) and the resulting solution was stirred for 15 min at r. t. Isocyanide (1 mmol) and carboxylic acid (1 mmol) were added and the stirring continued for 24 h. The crystalline precipitate (formed in case of products **5a**, **5i** and **5j**) was collected by filtration and washed with ether (3 ml); in all other cases, the solution was concentrated to dryness and the residue was purified by column chromatography on silica gel using dichloromethane-methanol (200:1) mixture as eluent.

#### 2.4.1. (E)-N-(2-(tert-Butylamino)-1–(4-methoxyphenyl)-2-oxoethyl)-N-isopropyl-4-oxo-4-(p-tolyl)but-2-enamide (4)

Yield 77 mg (17%); White amorphous solid; ^1^H NMR (400 MHz, CDCl_3_) δ 8.01 − 7.85 (m, 3H), 7.55 (d, *J* = 15.1 Hz, 1H), 7.35 − 7.30 (m, 4H), 6.90 (d, *J* = 8.8 Hz, 2H), 6.33 (s, 1H), 4.95 (s, 1H), 4.42 − 4.28 (m, 1H), 3.83 (s, 3H), 2.45 (s, 3H), 1.47 (d, *J* = 6.7 Hz, 3H), 1.35 (s, 9H), 1.17 (d, *J* = 6.8 Hz, 3H); ^13^C NMR (101 MHz, CDCl_3_) δ 189.0, 169.4, 165.9, 159.1, 144.7, 134.4, 134.2, 133.8, 129.5, 129.2, 129.0, 128.7, 114.1, 63.0, 55.2, 51.3, 50.4, 28.6, 21.7, 21.6, 21.6; HRMS (ESI), *m/z* calcd for C_27_H_34_N_2_O_4_ [M + Na]^+^ 473.2411, found 473.2398.

#### 2.4.2. (E)-N-(2-(tert-Butylamino)-1–(4-fluorophenyl)-2-oxoethyl)-N-cyclopropyl-4–(4-fluorophenyl)-4-oxobut-2-enamide (5)

Yield 97 mg (22%); Pale yellow amorphous solid; m.p.=115.5–117.1 °C; ^1^H NMR (400 MHz, CDCl_3_) δ 8.17 − 8.03 (m, 2H), 8.00 − 7.90 (m, 1H), 7.84 (d, *J* = 15.0 Hz, 1H), 7.48 − 7.38 (m, 2H), 7.24 − 7.14 (m, 2H), 7.13 − 7.05 (m, 2H), 5.79 (s, 1H), 5.64 (s, 1H), 2.74 − 2.52 (m, 1H), 1.40 − 1.29 (m, 9H), 1.21 − 1.05 (m, 1H), 1.02 − 0.91 (m, 1H), 0.85 − 0.65 (m, 2H); 13 C NMR (101 MHz, CDCl_3_) δ 188.0, 168.4, 167.6, 166.1 (d, *J* = 256.1 Hz), 162.5 (d, *J* = 248.5 Hz), 133.9, 133.8, 133.4 (d, *J* = 3.0 Hz), 131.6 (d, *J* = 9.5 Hz), 131.4 (d, *J* = 8.1 Hz), 131.2 (d, *J* = 3.4 Hz), 116.0 (d, *J* = 22.0 Hz), 115.6 (d, *J* = 21.5 Hz), 66.2, 51.6, 30.3, 28.6, 10.8, 9.2; HRMS (ESI), *m/z* calcd for C_25_H_26_F_2_N_2_O_3_ [M + Na]^+^ 463.1804, found 463.1794.

#### 2.4.3. (E)-2-((2-(tert-Butylamino)-1–(4-fluorophenyl)-2-oxoethyl)(methyl)amino)ethyl 4–(4-fluorophenyl)-4-oxobut-2-enoate (6)

Yield 55 mg (12%); Yellowish amorphous solid; ^1^H NMR (400 MHz, CDCl_3_) δ 8.08 (dd, *J* = 8.7, 5.4 Hz, 2H), 7.95 (d, *J* = 15.5 Hz, 1H), 7.36 − 7.25 (m, 2H), 7.23 (t, *J* = 8.6 Hz, 2H), 7.05 (t, *J* = 8.6 Hz, 2H), 7.01 − 6.87 (m, 2H), 4.37 (td, *J* = 5.9, 2.3 Hz, 2H), 3.94 (s, 1H), 2.81–2.63 (m, 2H), 2.28 (s, 3H), 1.37 (s, 9H); 13 C NMR (101 MHz, CDCl_3_) δ 187.5, 170.1, 166.2 (d, *J* = 256.8 Hz), 165.3, 162.4 (d, *J* = 246.8 Hz), 136.6, 132.9 (d, *J* = 3.0 Hz), 132.1, 131.8 (d, *J* = 3.4 Hz), 131.6 (d, *J* = 9.5 Hz), 130.6 (d, *J* = 8.0 Hz), 116.2 (d, *J* = 22.0 Hz), 115.3 (d, *J* = 21.5 Hz), 75.1, 62.7, 53.3, 50.8, 40.1, 28.6; HRMS (ESI), *m/z* calcd for C_25_H_28_F_2_N_2_O_4_ [M + Na]^+^ 481.1915, found 481.1893.

### 2.5. N,N-Bis(2,4-dimethoxybenzyl)-4-formylbenzenesulphonamide (12)

A solution of 4-formylbenzenesulphonyl chloride^14^ (195 mg, 0.95 mmol), bis(2,4-dimethoxybenzyl)amine (302 mg, 0.95 mmol) and trimethylamine (193 mg, 1.91 mmol) in dichloromethane (10 ml) was stirred for 30 min at room temperature and then washed with 10% aq. HCl (10 ml), saturated NaHCO_3_ (2 × 10 ml) and brine (10 ml). The resulting solution was evaporated to dryness. Yield 430 mg, 93%; Yellow oil; ^1^H NMR (400 MHz, CDCl_3_) δ 10.08 (s, 1H), 7.99 − 7.84 (m, 2H), 7.79 (d, *J* = 8.3 Hz, 2H), 7.18 (d, *J* = 8.4 Hz, 2H), 6.41 (dd, *J* = 8.4, 2.4 Hz, 2H), 6.27 (d, *J* = 2.4 Hz, 2H), 4.45 (s, 4H), 3.79 (s, 6H), 3.62 (s, 6H); 13 C NMR (101 MHz, CDCl_3_) δ 191.0, 160.5, 158.2, 146.7, 138.0, 130.9, 129.6, 127.5, 116.6, 104.0, 98.0, 55.4, 54.9, 46.5.

### 2.6 (E)-N-(2-(tert-butylamino)-2-oxo-1–(4-sulphamoylphenyl) ethyl)-N-cyclopropyl-4-oxo-4-(p-tolyl)but-2-enamide (10)

A solution of *N*,*N*-bis(2,4-dimethoxybenzyl)−4-formylbenzenesulphonamide (97 mg, 0.2 mmol) and cyclopropylamine (11 mg, 0.2 mmol) in 2 ml of methanol was stirred for 15 min at room temperature, then *tert*-butyl isocyanide (17 mg, 0.2 mmol) and (*E*)−4-oxo-4-(*p*-tolyl)but-2-enoic acid (38 mg, 0.2 mmol) were added to the solution and the resulting mixture was stirred for 24 h at the same temperature. The solvent was removed under reduced pressure and the residue was purified by column chromatography using eluent ethyl acetate–hexane 1:1 (R_f_=0.25). The Ugi adduct **13** thus obtained was dissolved in trifluoroacetic acid (3 ml) and the solution was stirred for 3 h. The solvent was evaporated and the residue was redissolved in 3 ml of methanol, filtered from insoluble impurities and evaporated. White semi-solid; Yield 12 mg, 12%; ^1^H NMR (400 MHz, Methanol-*d*_4_) δ 7.98 (d, *J* = 8.0 Hz, 2H), 7.96 − 7.91 (m, 2H), 7.87 (d, *J* = 15.3 Hz, 1H), 7.81 (d, *J* = 15.3 Hz, 1H), 7.56 (d, *J* = 8.2 Hz, 2H), 7.39 (d, *J* = 7.9 Hz, 2H), 5.90 (s, 1H), 2.78 − 2.55 (m, 1H), 2.45 (s, 3H), 1.38 (s, 9H), 1.16 − 1.04 (m, 1H), 0.83 (ddd, *J* = 9.6, 4.8, 2.2 Hz, 1H), 0.78 − 0.67 (m, 1H), 0.58 − 0.45 (m, 1H); 13 C NMR (101 MHz, Methanol-*d*_4_) δ 189.6, 169.0, 168.5, 145.0, 143.3, 140.1, 134.4, 133.8, 133.6, 130.0, 129.3, 128.7, 125.8, 65.3, 51.1, 29.3, 27.4, 20.3, 10.3, 9.0; HRMS (ESI/Q-TOF) *m/z*: [M + Na]^+^ Calcd for C_26_H_31_N_3_O_5_S 520.1877; Found 520.1874.

### 2.3. Cell viability assay

Human cell lines were maintained at 37 °C in humidified atmosphere containing air and 5% CO_2_ as previously described.^15^ Pancreas ductal adenocarcinoma cells PANC-1 were obtained from Russian collection cell cultures at the RAS Institute of Cytology (Saint Petersburg, Russian Federation). Cell line were grown in Dulbecco’s Modified Eagle’s Medium-F12 (BioloT) containing 10% (v/v) heat-inactivated foetal calf serum (FCS, HyClone Laboratories, UT), 1% L-glutamine, 1% sodium pyruvate, 50 U/ml penicillin, and 50 μg/ml streptomycin (BioloT). Cytotoxicity of carbonic anhydrase inhibitors was evaluated using a routine colorimetric method with tetrazolium dye − 3–(4,5-dimethylthiazol-2-yl)−2,5-diphenyltetrazolium bromide (MTT). The cell lines were incubated for 48 h under normoxia and in the presence of the hypoxia-mimicking agent 50 μM CoCl_2_ with medium containing different concentrations of carbonic anhydrase inhibitors. Following treatment, Dulbecco’s Modified Eagle’s Medium-F12 (100 μl/well) and 20 μl of a 2.5 mg/ml MTT solution were added and cells were incubated for 1 h at 37 °C. The used cell density was 5 × 103 cells/200 μl/well in 96-well microtiter plates. After aspiration of the supernatants, the MTT-formazan crystals formed by metabolically active cells were dissolved in dimethyl sulphoxide (100 μl/well) and absorbance was measured at 540 nm and 690 nm in Varioskan LUX™ Multimode Microplate Reader (Thermo Scientific, Waltham, MA). Values measured at 540 nm were subtracted for background correction at 690 nm, and the data were plotted as a percent of control untreated samples.

## 3. Results and discussion

### 3.1. Chemistry

**Scheme 1.**
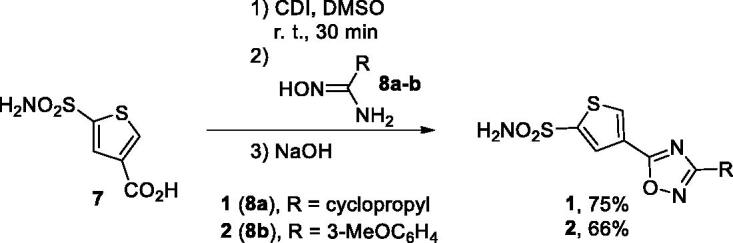
Preparation of compounds **1** and **2**.

Compounds **1** and **2** were synthesised from common precursor − 5-sulphamoylthiophene-3-carboxylic acid (**7**) as described previously.^5^ Carboxylic acid **7** was activated by treatment with CDI and the resulting imidazolide was reacted with amidoximes **8a-b**. Addition of solid NaOH formed a superbase solution in DMSO which triggered cyclodehydration yielding compounds **1** and **2** in good yields (Scheme 1).

**Scheme 2.**
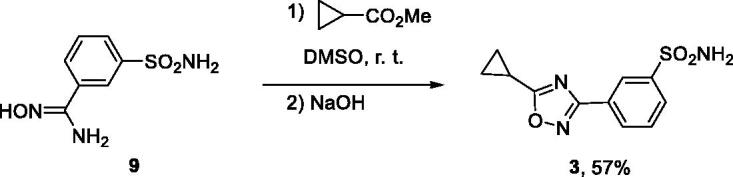
Preparation of compound **3**.

Isomeric 1,2,4-oxadiazole **3** was prepared also as described previously.^5^ Amidoxime **9** was acylated with methyl cyclopropanecarboxylate in DMSO whereupon addition of solid NaOH, again, led to cyclodehydration to give compound **3** in 57% yield (Scheme 2).

**Scheme 3.**
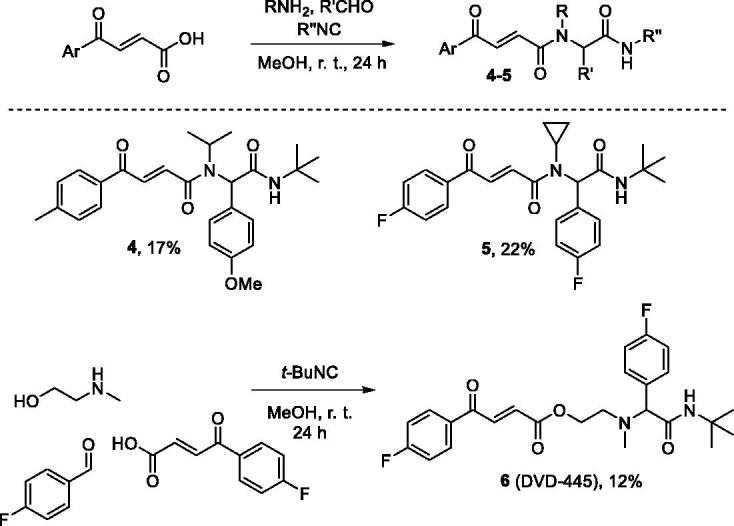
Preparation of compounds **4**–**6**.

Diamides **4** and **5** were prepared *via* the traditional Ugi reaction. However, employing secondary b-(methylamino)ethanol as the amine component in the preparation of compound **6** (DVD-445) produced a different, amide ester scaffold (Scheme 3).^12^

**Scheme 4.**
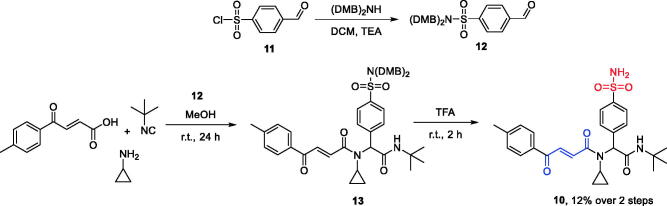
Preparation of compound **10**.

For the synthesis of UMA/primary sulphonamide ZBG hybrid **10**, the following synthetic strategy was adopted. Known^14^ sulphonyl chloride **11** was converted to bis-DMB-protected (DMB = 2,4-dimethoxybenzyl) aldehyde **12**. The latter was involved in the Ugi reaction with cyclopropylamine, *tert*-butyl isocyanide, and *p*-toluoyl acrylic acid conducted at ambient temperature in methanol to give adduct **13** which was used in the next step without further purification. Removal of the DMB protecting groups with TFA gave the target UMA/ZBG hybrid **10** in modest yield (Scheme 4).

### 3.2. Biological evaluation

**Figure 1.**
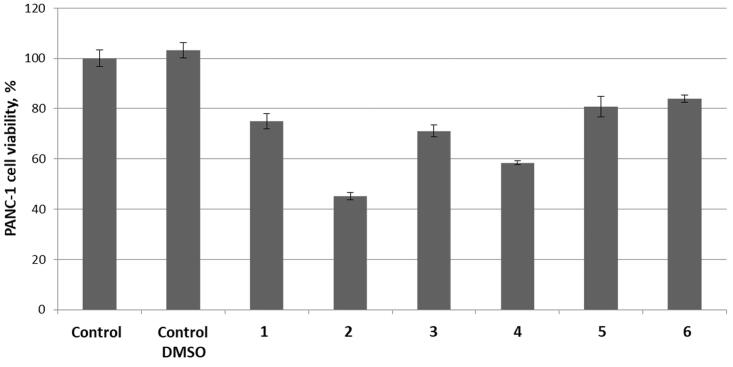
Cytotoxicity of compounds **1**–**3** (100 μM) and **4**–**6** (1 μM) as single agents against PANC-1 cell line.

Initially, compounds **1**–**3** (100 μM) and **4**–**5** (1 μM) were tested as single agents against pancreas ductal adenocarcinoma cell line (PANC-1) to establish the basal cytotoxicity levels of the two groups of compounds exerting their cytotoxicity *via* entirely different mechanisms (*h*CA IX/XII inhibition and TrxR inhibition, respectively). As the result, only compounds **2** and **4** had a pronounced antiproliferative effect reducing the cell viability by >50% and >40%, respectively while the other four compounds (**1**, **3**, **5**–**6**) were only marginally cytotoxic at these concentrations (Figure 1).

**Figure 2.**
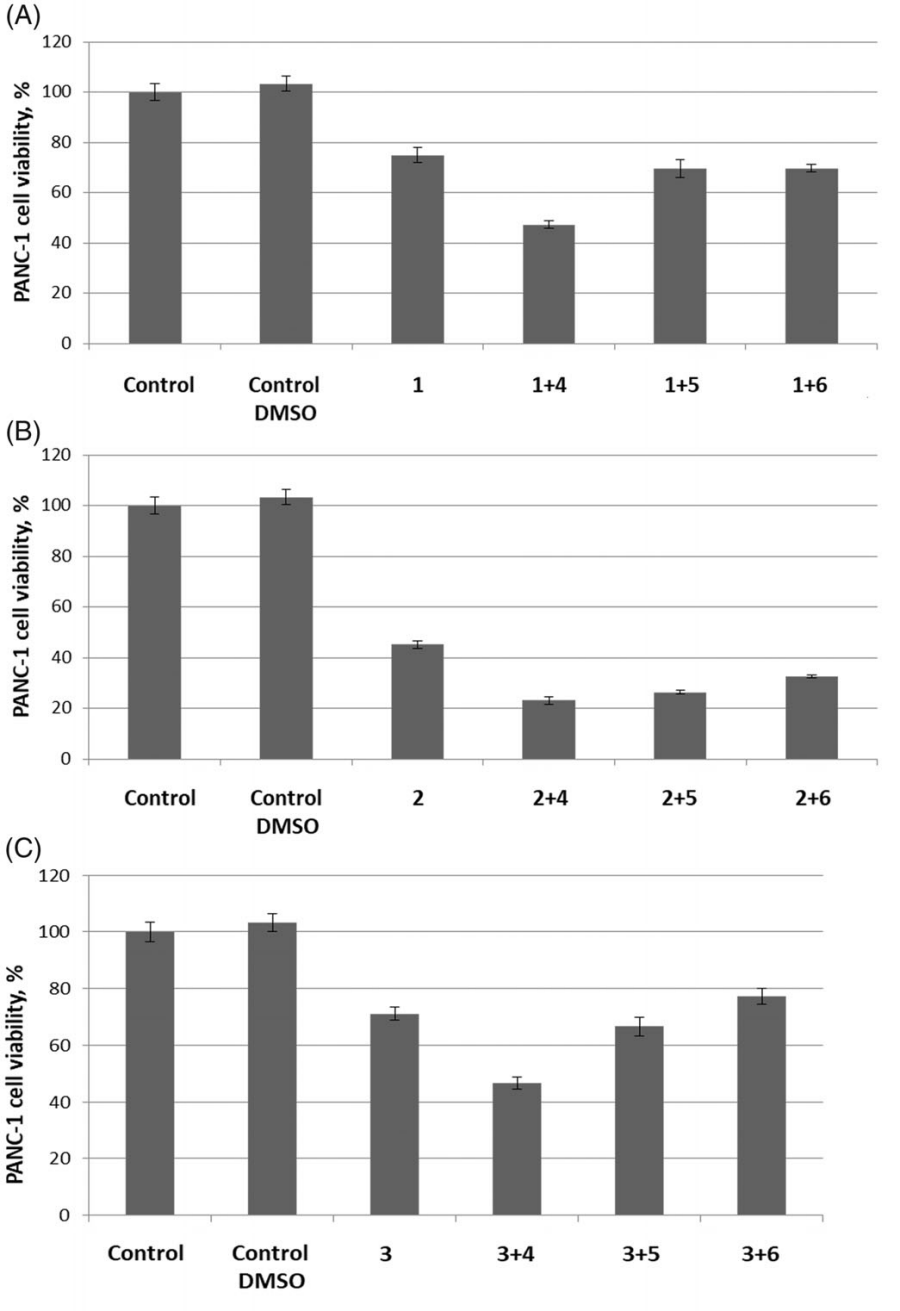
Cytotoxicity towards PANC-1 cell line of carbonic anhydrase inhibitors **1** (A), **2** (B) and **3** (C) (100 μM) tested in combination with 1 μM concentrations of TrxR inhibitors **4**, **5,** or **6**.

Next, we proceeded to text carbonic anhydrase inhibitors **1**–**3** (100 μM) in combination with thioredoxin reductase inhibitors **4**–**6** (1 μM). To our delight, in all cases, TrxR inhibitor **4** produced a strong potentiation of the carbonic anhydrase inhibitor’s antiproliferative activity. Addition of TrxR inhibitors **5** and **6** seemed to make little difference for the antiproliferative effect of carbonic anhydrase inhibitors **1** and **3**. However, all three TrxR inhibitors **4**–**6** noticeably potentiated the cytotoxicity of carbonic anhydrase inhibitor **2** (Figure 2).

**Figure 3.**
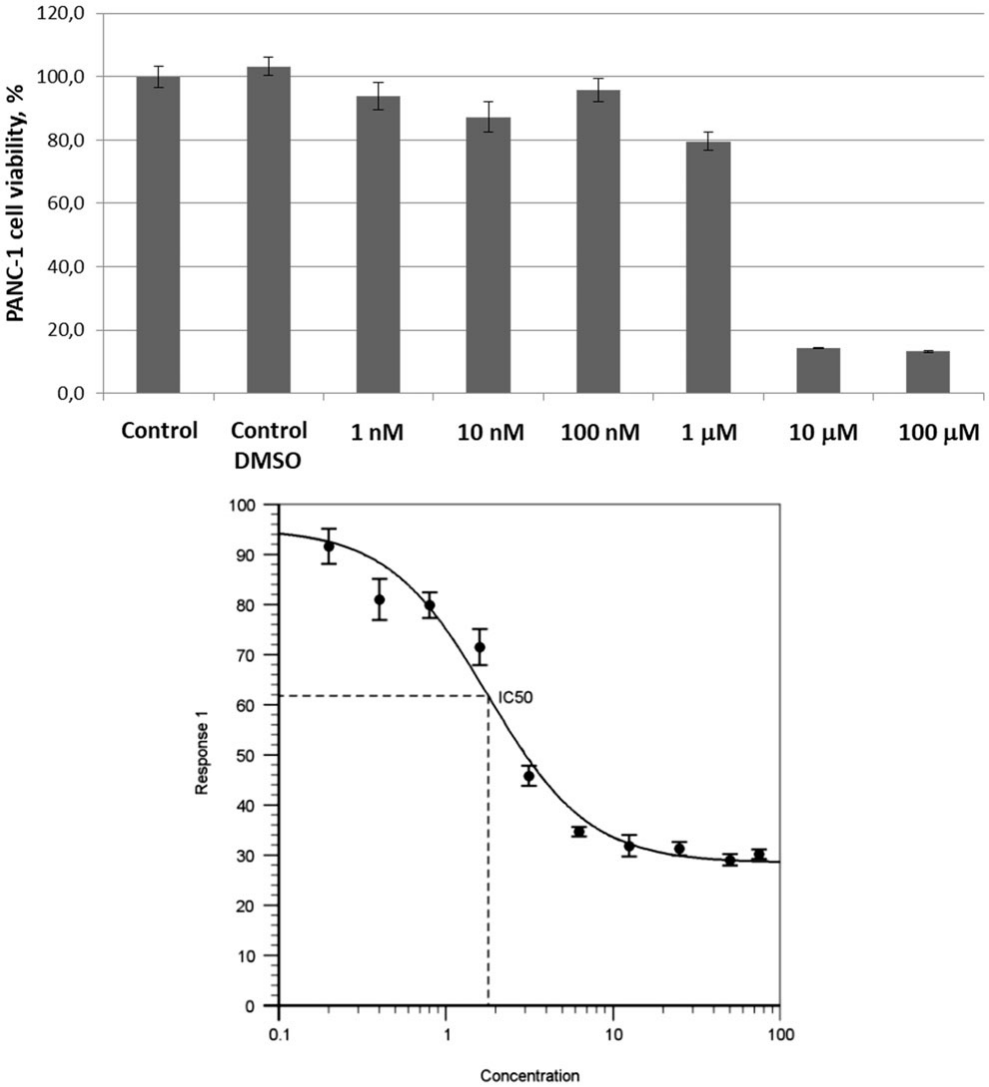
Cytotoxicity of compound **10** towards PANC-1 cell line.

Encouraged by these findings, we also tested UMA/ZBG hybrid **10** against the same cell line. Pre-emptively expecting higher cytotoxicity of this compound compared to **1**–**3** and **4**–**5** taken as single agents or even in combination with each other, we tested compound **10** in the 1 − 100 μM concentration range. On increasing the concentration of compound **10** from 1 to 10 μM a sharp decrease in the number of viable cells was observed. To confirm this cytotoxic effect being dose-dependent, a full dose–response curve was obtained from which an IC_50_ value of 1.8 ± 0.4 μM was deduced (Figure 3).

## 4. Conclusions

We hypothesised that simultaneous targeting of two cancer cells’ defence mechanism – those against hypoxia and oxidative stress – by cooperative inhibiting carbonic anhydrase IX/XII and thioredoxin reductase, respectively, would have a synergistic effect. This was confirmed by testing several combinations of the previously reported CA IX/XII and TrxR inhibitors. These results led to an idea of combining CA and TrxR inhibitory pharmacophores (a primary sulphonamide zinc-binding groups and a Michael acceptor moiety, respectively) in the structure of a single agent. Testing a pilot compound conforming to this design principle demonstrated that the compound has a pronounced cytotoxic effect against pancreas ductal adenocarcinoma cells PANC-1 (IC_50_ = 1.8 ± 0.4 μM). This observation validated a new approach to the design of potential anticancer agents. Further studies are underway in our laboratories which are aimed at further investigating the structure-activity relationships of structurally diverse zinc-binding group-containing Michael acceptors as antiproliferative agents and to correlate the phenotypic assay data with the inhibition profile against the isolated recombinant enzymes. The results of these studies will be reported in due course.

Detailed experimental procedures for synthesis, complete characterization data and copies of ^1^H and ^13 ^C NMR spectra are available online (Supplementary material).

## Supplementary Material

Supplemental Material
