# Supplementary material for: Combining carbonic anhydrase and thioredoxin reductase inhibitory motifs within a single molecule dramatically increases its cytotoxicity
Source: J Enzyme Inhib Med Chem. 2020 Mar 5;35(1):665–71. doi: 10.1080/14756366.2020.1734800 (PMC7067156; doi:10.1080/14756366.2020.1734800)

Supplementary Information for

Combining carbonic anhydrase and thioredoxin reductase inhibitory motifs within a single molecule dramatically increases its cytotoxicity

Mikhail Krasavin[a,*], Tatiana Sharonova[a], Vladimir Sharoyko[a], Daniil Zhukovsky[a], Stanislav Kalinin[a], Raivis Žalubovskis[c,d], Tatiana Tennikova[a], and Claudiu T. Supuran[d,*]

Contents

Copies of ^{1}H and ^{13}C NMR spectra of compounds **12** and **10** 2-3

^{1}H and ^{13}C NMR spectra of compound 12 ($CDCl_3$)

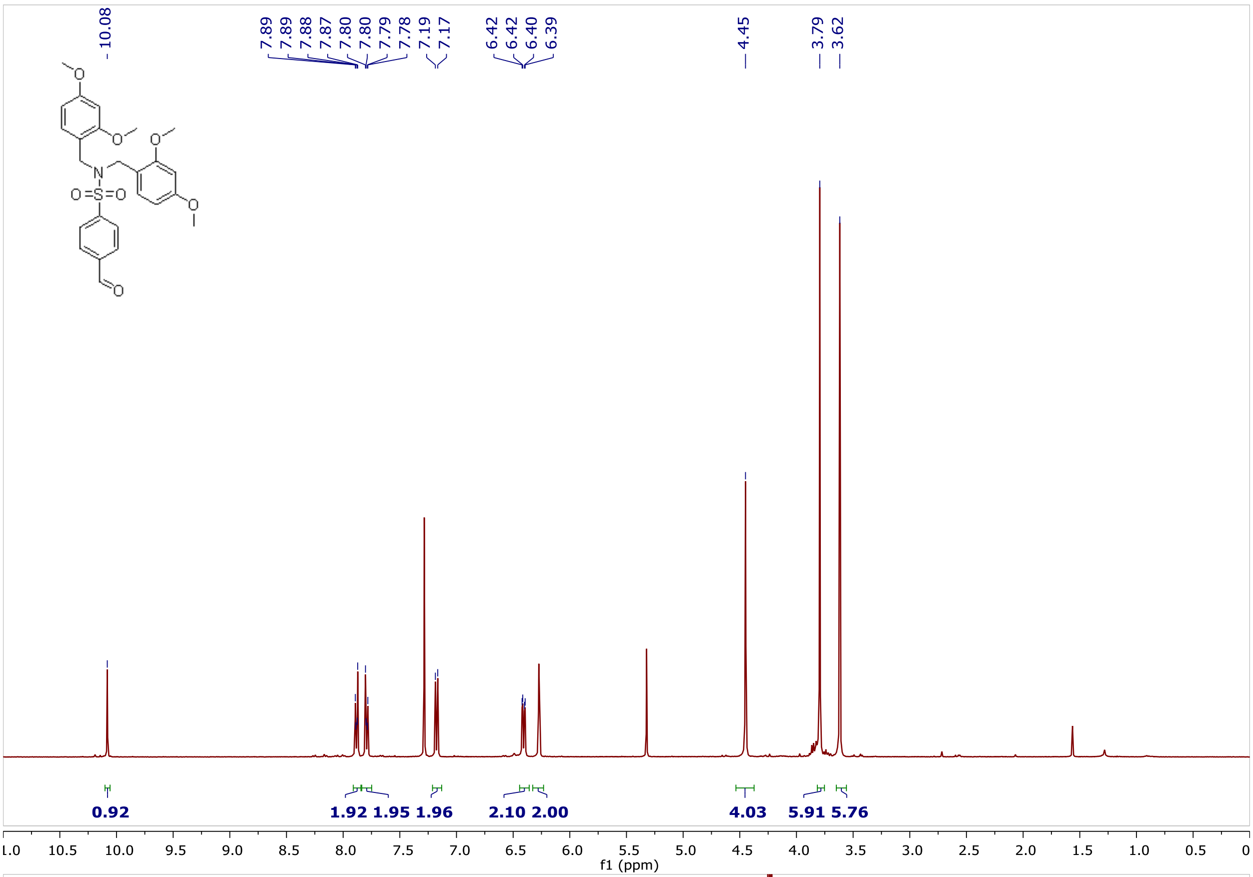

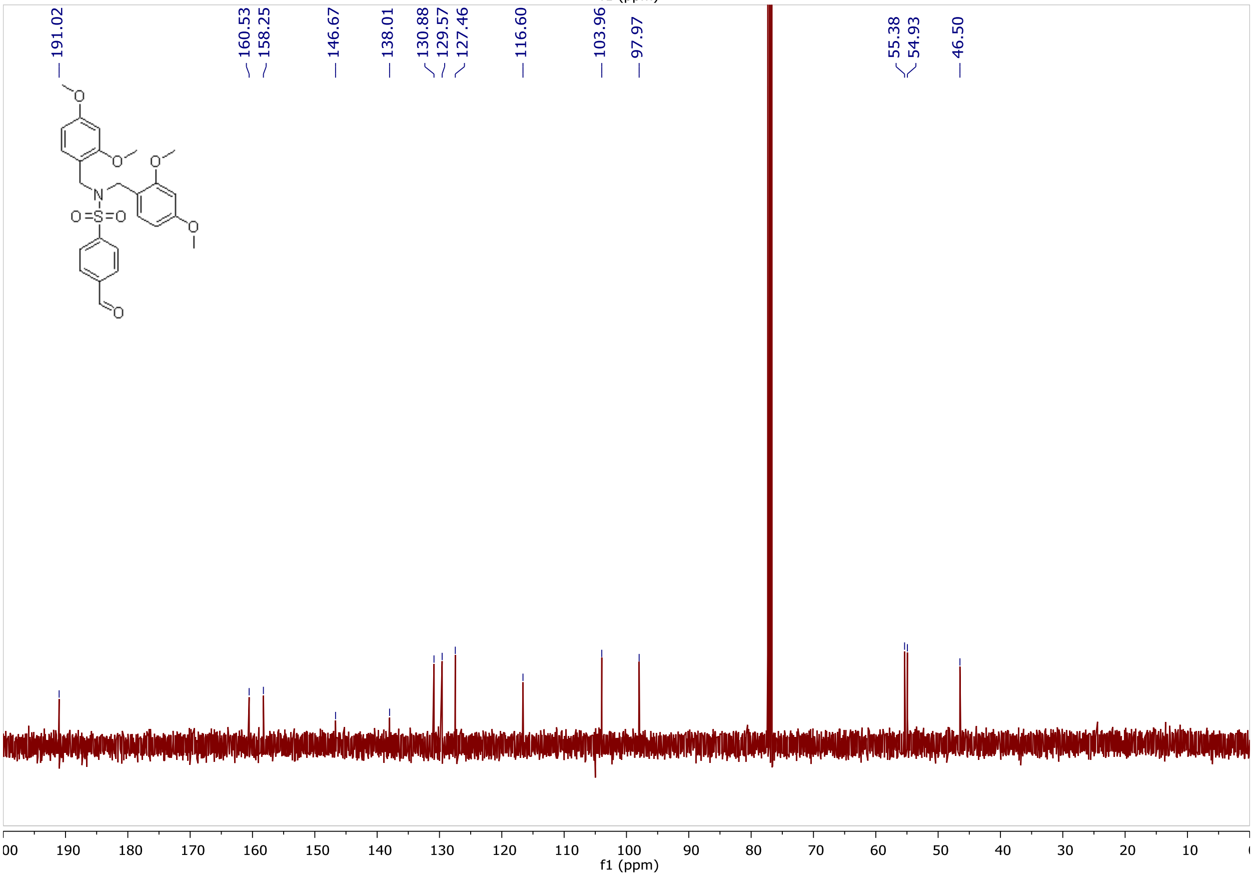

^{1}H and ^{13}C NMR spectra of compound 10 (CD_3OD)

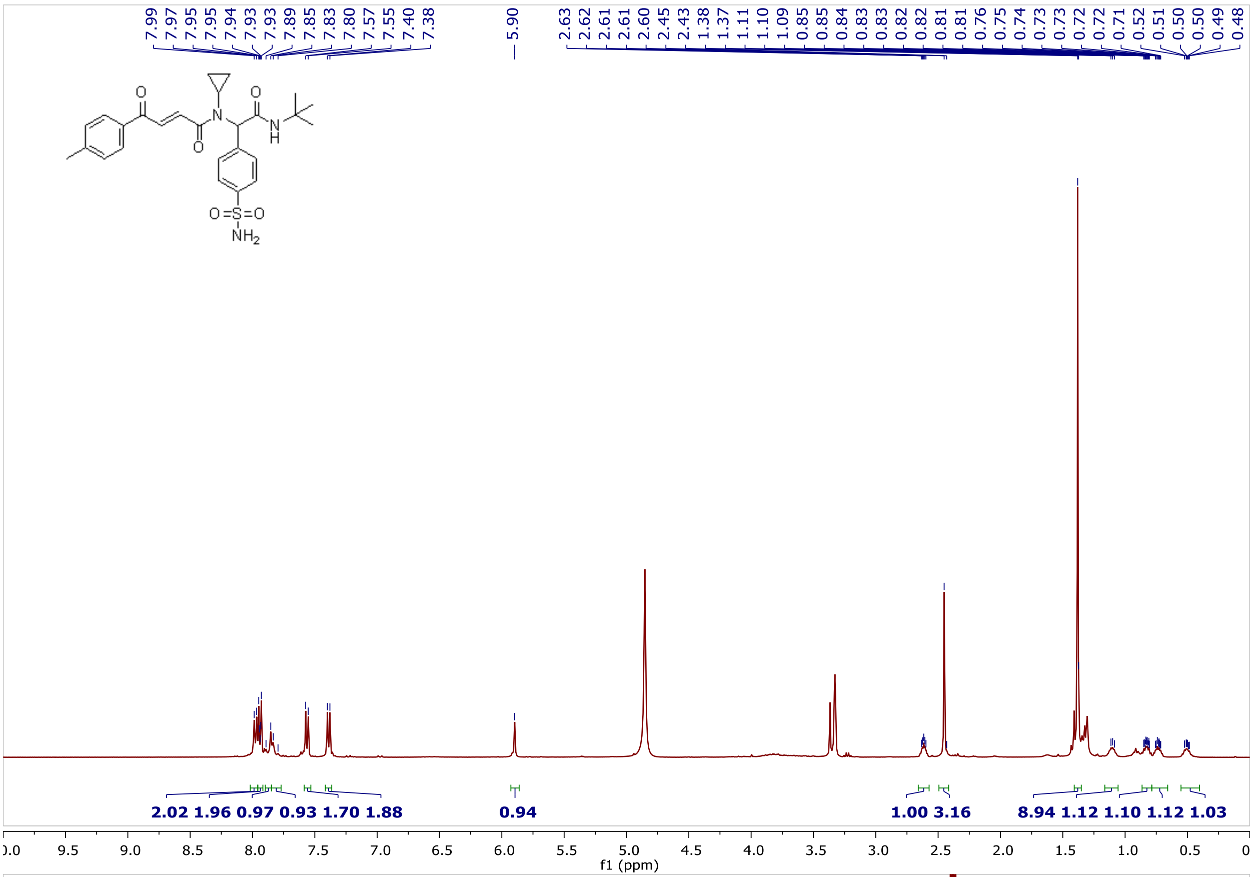

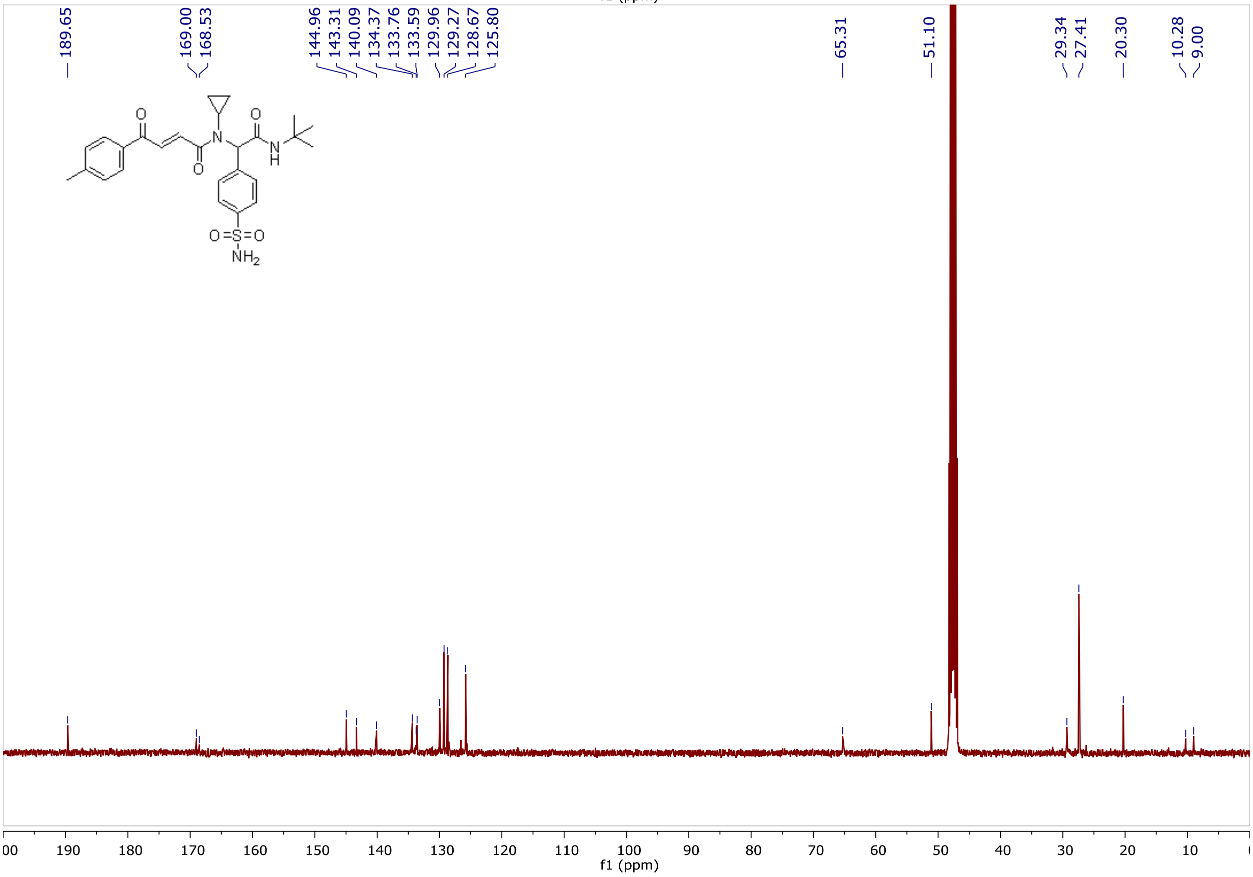

Supplement: Supplemental Material [file IENZ_A_1734800_SM8666.pdf]
